# Hypomethylation of ERVs in the sperm of mice haploinsufficient for the histone methyltransferase Setdb1 correlates with a paternal effect on phenotype

**DOI:** 10.1038/srep25004

**Published:** 2016-04-26

**Authors:** Lucia Daxinger, Harald Oey, Luke Isbel, Nadia C. Whitelaw, Neil A. Youngson, Alex Spurling, Kelly K. D. Vonk, Emma Whitelaw

**Affiliations:** 1 Department of Genetics, La Trobe Institute for Molecular Science, La Trobe University, Bundoora, VIC, 3086 Australia; 2 Department of Human Genetics, Leiden University Medical Centre, Leiden, The Netherlands; 3 Epigenetics Laboratory, QIMR Berghofer Medical Research Institute, Herston, Brisbane 4006, QLD, Australia; 4 Department of Pharmacology, School of Medical Sciences, UNSW Australia, Sydney 2052, Australia

## Abstract

The number of reports of paternal epigenetic influences on the phenotype of offspring in rodents is increasing but the molecular events involved remain unclear. Here, we show that haploinsufficiency for the histone 3 lysine 9 methyltransferase Setdb1 in the sire can influence the coat colour phenotype of wild type offspring. This effect occurs when the allele that directly drives coat colour is inherited from the dam, inferring that the effect involves an “*in trans*” step. The implication of this finding is that epigenetic state of the sperm can alter the expression of genes inherited on the maternally derived chromosomes. Whole genome bisulphite sequencing revealed that *Setdb1* mutant mice show DNA hypomethylation at specific classes of transposable elements in the sperm. Our results identify *Setdb1* as a paternal effect gene in the mouse and suggest that epigenetic inheritance may be more likely in individuals with altered levels of epigenetic modifiers.

Epigenetic modifications that influence gene expression patterns and thereby contribute to the phenotype of an organism are usually cleared on passage through the germline. This is to ensure the totipotency of the zygote and also serves to erase environmentally-acquired epigenetic states and/or epigenetic errors that have arisen in the parental generation. An increasing number of reports that epigenetic states can be inherited to the next generation via the gametes challenge this dogma and are of particular interest because they alter our understanding of the inheritance of phenotypic traits^1^,^2^,^3^,^4^,^5^,^6^,^7^,^8^,^9^,^10^,^11^,^12^,^13^. In mammals, transgenerational epigenetic inheritance has been reported at the *Agouti viable yellow* (*A*^*vy*^) allele in the mouse^14^,^15^. Since this discovery, the *A*^*vy*^ allele has been extensively used as an epigenetically sensitive reporter to investigate the contribution of genetic and environmental influences to epigenetic state both within and across generations^16^,^17^,^18^,^19^,^20^,^21^,^22^,^23^. However, little is known about the factors that determine the epigenotype at *A*^*vy*^.

Two genes that show a paternal effect at *A*^*vy*^, *Dnmt1* and *Snf2h/Smarca5*, have been described^24^. In other words, heterozygosity for mutations in Dnmt1 or Snf2h/Smarca5 in the sire, can affect the coat colour of offspring that do not inherit the mutant alleles. The paternal effect must occur *in trans* because the *A*^*vy*^ allele is inherited via the wild type female^24^. Similar effects of father-to-offspring transmission have been reported for heat-shock-induced epigenetic memory^9^ and paternal obesity^2^ in *Drosophila*, as well as Dnmt3l-dependent sex chromosome polyploidy^24^, olfaction-dependent behavioural and neural phenotypes^5^ and metabolic control^3^,^1^,^4^ in the mouse. Some of them involve *in trans* mechanisms^2^,^9^,^24^. Unravelling the underlying molecular events has remained difficult and identification of the genetic factors required for these processes is needed.

Expression of the *A*^*vy*^ locus is driven by an intracisternal A particle (IAP) retrotransposon, a member of the ERVK family of repeat elements, that has inserted upstream of the agouti gene^25^. A cryptic promoter within the 3′ long terminal repeat (LTR) of the IAP directs transcription of the agouti coding exons^14^,^25^,^26^. The coat colour in isogenic littermates varies from yellow through to mottled to pseudoagouti (brown) and correlates with the level of DNA methylation at the LTR promoter^14^. DNA methylation was the first epigenetic mark recognized to contribute to ERV silencing^27^,^28^. However, it has now become clear that in mouse embryonic stem cells (mESCs) and primordial germ cells (PGCs) IAPs are enriched for the repressive H3 lysine 9 trimethylation (H3K9me3) and two factors, the H3K9 methyltransferase Setdb1 (SET-domain bifurcated 1, also known as ESET) and the transcriptional co-repressor Trim28 (Tripartite motif-containing 28, also known as Kap1 or Tif1b) have been shown to play essential roles in the silencing of these elements^29^,^30^,^31^. We hypothesized that *Setdb1* and *Trim28* might be critical factors for establishment of epigenetic state and epigenetic inheritance at *A*^*vy*^.

Here, we report a paternal effect as a result of reduced levels of Setdb1 on expression of a maternally inherited *A*^*vy*^ allele. In contrast, a similar effect is not observed if the reduced levels of Setdb1 occur in the dam. We find that ERVK retrotransposons are hypomethylated in the sperm of heterozygous Setdb1 mutant mice. We also show that Trim28 is required for establishment of epigenetic state at *A*^*vy*^ but haploinsufficiency of the sire has no influence on coat colour phenotype of the wild type offspring. In summary our data suggests that dosage of some epigenetic modifiers is more critical in male gametes than female gametes, providing new insights into the mechanisms underlying epigenetic inheritance in mammals.

## Results

### Experimental design and coat colour phenotype

We have recently reported the identification of a number of *Modifiers of murine metastable epialleles dominant* (*MommeD*) mouse lines^32^. These mutant lines were identified in an *N-ethyl-N-nitrosourea* (ENU) mutagenesis screen for factors required for epigenetic reprogramming in the mouse^33^ and can be used to study the effect of *MommeD* genes on epigenetic inheritance at *A*^*vy*^^24^,^32^,^34^.

**Figure 1:**
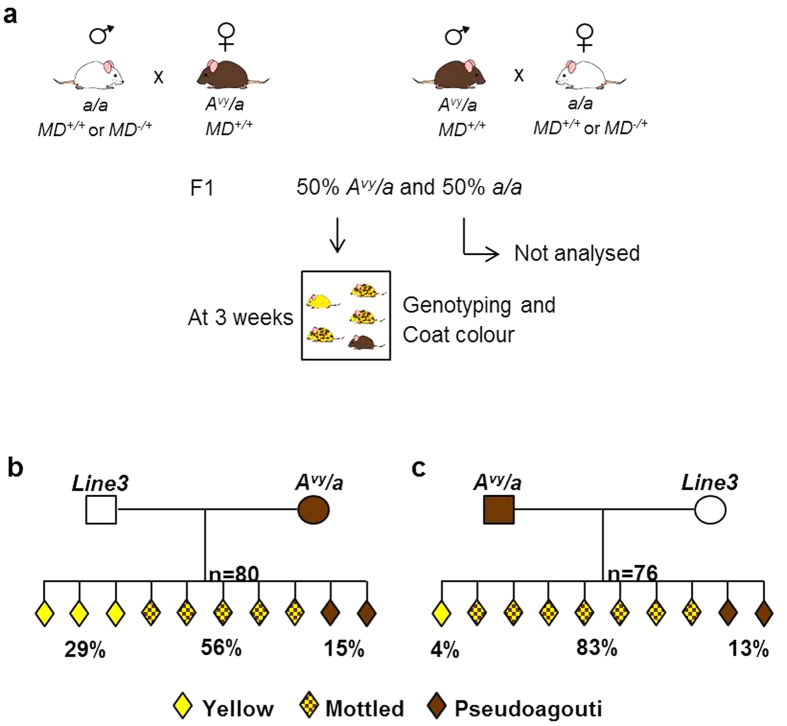
Pedigrees of control crosses between Line3 and mice carrying the *A*^*vy*^ allele. (**a**) Schematic of experimental setup of *A*^*vy*^ crosses. (**b**) Coat colour of offspring from crosses between wild type (Line3) sires and pseudoagouti (*A*^*vy*^) dams. (**c**) Coat colour of offspring from crosses between pseudoagouti (*A*^*vy*^) sires and wild type (Line3) dams. Data were produced from at least five different mating pairs in each case. Offspring not carrying the *A*^*vy*^ allele have been omitted.

We carried out reciprocal crosses between FVB/N (Line3) mice heterozygous for a *MommeD* mutation (*MD*^−/+^) and pseuodagouti (brown) C57BL/6 mice heterozygous for the *A*^*vy*^ allele (*A*^*vy*^*/a*) (Fig. 1a). In parallel, we set up control reciprocal crosses between wild type FVB/N (Line3) (*MD*^+/+^) mice and pseudoagouti C57BL/6 mice heterozygous for the *A*^*vy*^ allele (*A*^*vy*^*/a*) (Fig. 1a). FVB/N (Line3) mice carry a wild type agouti locus, called *A* that is not sensitive to epigenetic state. C57BL/6 mice are homozygous for the null allele, called *a*, hence their black coat. The offspring were all C57/FVB (Line3) F1 hybrids and differed only at the *MD* and *A*^*vy*^ locus. Offspring were scored, at weaning, for the coat colour phenotype and genotyped for the presence of the *A*^*vy*^ allele. Importantly, only animals with an *A/a* genotype were included in the analysis and all of these were genotyped for the *MommeD* mutation. Because the parental mice used were heterozygous for the *MommeD* mutation, both wild type and mutant offspring were produced.

When the *A*^*vy*^ allele was inherited from the dam, the control cross produced offspring with a range of coat colour phenotypes; 29% yellow, 56% mottled and 15% pseudoagouti (Fig. 1b). When the *A*^*vy*^ allele was inherited from the male, the control cross produced offspring with the following range of coat colours; 4% yellow, 83% mottled and 13% pseudoagouti (Fig. 1c). It is known that penetrance at *A*^*vy*^ is affected by parent of origin^14^,^15^ and we have previously demonstrated that the paternally and maternally inherited alleles are treated differently during epigenetic reprogramming^34^.

### *Setdb1* ^*MD13*^ shows a paternal effect on expression of *A* ^*vy*^

The *Setdb1*^*MD13*^ allele has a splice site mutation that introduces a premature stop codon^32^. Heterozygotes are haploinsufficient for Setdb1 protein (Supplementary Fig. 1) and are viable and fertile^32^. Similarly, the *Trim28*^*MD9*^ allele is a null and mutant mice are haploinsufficient for Trim28 protein. *Trim28*^*MD9*^ heterozygotes show behavioural abnormalities, stochastic obesity and females have decreased fertility^35^.

**Figure 2:**
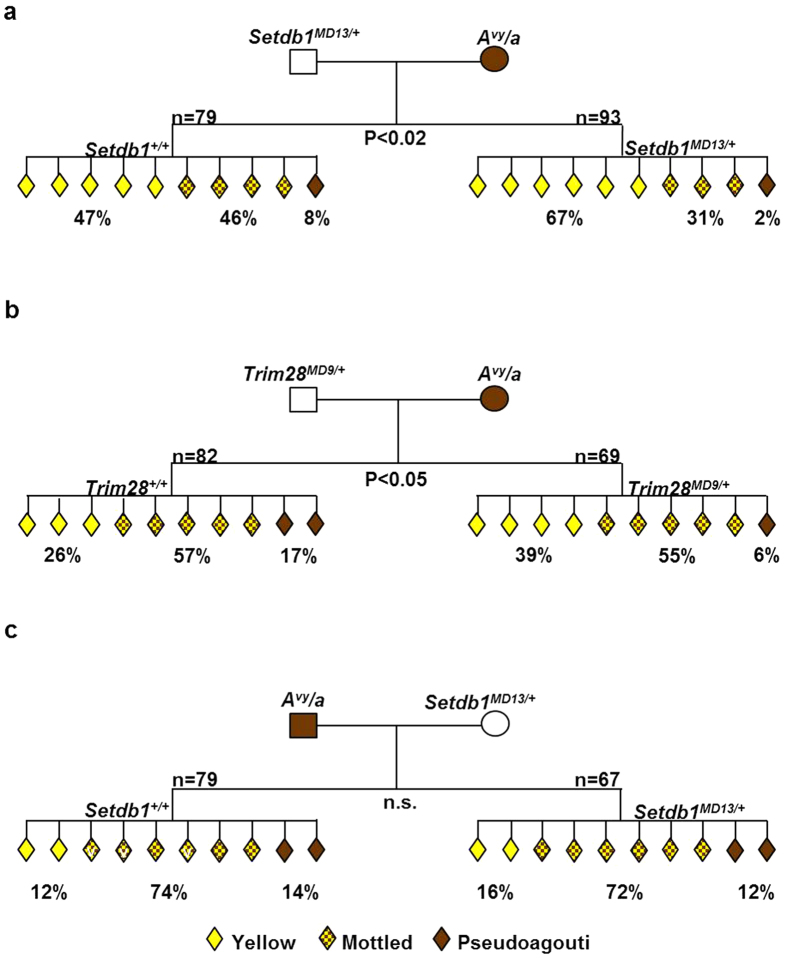
Pedigrees of crosses between *Setdb1*^*MD13*^ or *Trim28*^*MD9*^ and mice carrying the *A*^*vy*^ allele. **(a**) Pedigree produced from a *Setdb1*^*MD13*/+^ sire and pseudoagouti (*A*^*vy*^) dam. Wild type (WT) offspring from *Setdb1*^*MD13*/+^ sires showed a significant increase (Chi-square test: P < 0.001) in the proportion of animals with yellow coats when compared with WT offspring from WT sires. Offspring heterozygous for *Setdb1*^*MD13*^ demonstrated a significant shift in penetrance toward yellow when compared to WT littermates (Chi-square test: P < 0.02). (**b**) Pedigree produced from a *Trim28*^*MD9*/+^ sire and pseudoagouti (*A*^*vy*^) dam. *Trim28*^*MD9*^ heterozygotes exhibited a significant shift in penetrance toward yellow when compared with WT littermates (Chi-square test: P < 0.05). (**c**) Pedigree produced from a pseudoagouti (*A*^*vy*^) sire and *Setdb1*^*MD13*/+^ dam. Offspring showed no shift in penetrance at *A*^*vy*^. For all crosses data were produced from at least five different mating pairs. Offspring not carrying the *A*^*vy*^ allele have been omitted.

To test if Setdb1 haploinsufficiency in the sire affects expression of *A*^*vy*^ we crossed *Setdb1*^*MD13*/+^ males with pseudoagouti (*A*^*vy*^/a) females and analysed offspring for coat colour. First, we compared wild type to mutant offspring and found that offspring that inherited the *Setdb1*^*MD13*^ allele were more likely to be yellow (67% yellow) than their wild type littermates (47% yellow, Chi-squared test < 0.02) (Fig. 2a). In other words, haploinsufficiency for Setdb1 decreased the probability of silencing at the *A*^*vy*^ locus. These findings indicate that Setdb1 is involved in silencing of *A*^*vy*^ in the early embryo.

When we compared the coat colour of wild type offspring from the control cross with the coat colour of wild type offspring (*Setdb1*^+/+^) from the *Setdb1*^*MD13/*+^ males we discovered a paternal effect; the coat colours of wild type offspring produced from a *Setdb1*^*MD13*/+^ sire were more likely to be yellow (47% yellow) than the coat colours of wild type mice from wild type sires (29% yellow, Chi-squared test < 0.001) (Figs 2a and 1c). Importantly, the offspring in both groups were genetically identical and only differed with respect to the untransmitted genotype of the sire. Because the *A*^*vy*^ locus is transmitted by the dam (this locus is not present in the heterozygous *Setdb1*^*MD13*^ sire) the “*in trans”* paternal effect must be a consequence of events in the *Setdb1*^*MD13*^ mutant sire.

We also crossed males heterozygous for *Trim28*^*MD9*^ with pseudoagouti (*A*^*vy*^/a) females and analysed the offspring for coat colour. Firstly, we found that offspring heterozygous for *Trim28*^*MD9*^had an increased probability of being yellow (39%) compared to wild type littermates (29%, Chi-squared test < 0.05) (Fig. 2b), i.e. Trim28 is involved in silencing at the *A*^*vy*^ allele. In this case we did not see a paternal effect, i.e. wild type offspring from wild type sires and wild type offspring from *Trim28*^*MD9*^ sires displayed a similar range of coat colour (Figs 2b and 1c).

Our data provides genetic evidence that normal levels of both Setdb1 and Trim28 are required for the establishment of epigenetic state at *A*^*vy*^ in the early post implantation embryo, when the *A*^*vy*^ allele is inherited maternally. Most likely this occurs between the blastocyst stage and gastrulation when epigenetic state at *A*^*vy*^ is established^34^. This suggests a requirement of both Setdb1 and Trim28 for silencing of IAPs in the early embryo. Interestingly, the dosage effect on the *A*^*vy*^ phenotype is more pronounced for *Setdb1*^*MD13*^ than *Trim28*^*MD9*^ (i.e. there is a greater fraction of yellow offspring in heterozygotes of the former than the latter) (Fig. 2a,b). This may be due to a greater sensitivity to reduced levels of Setdb1 than Trim28 in the early embryo when epigenetic state at *A*^*vy*^ is established. Alternatively, this could result from the compounding of two separate events, first, the paternal effect that occurs after fertilization and is the result of an altered epigenetic state of *Setdb1*^*MD13*^ sperm and second, the requirement of Setdb1 for establishment of epigenetic state at *A*^*vy*^ in the early embryo.

### Maternal Setdb1 haploinsufficiency has no effect on expression of *A* ^*vy*^

Next, we tested if a paternally inherited *A*^*vy*^ allele is sensitive to maternal haploinsufficiency for Setdb1. We crossed females heterozygous for *Setdb1*^*MD13*^ with male pseudoagouti *A*^*vy*^/a mice and scored offspring for coat colour. We did not see a shift in the coat colour ratios of wild type or mutant offspring (Figs 2c and 1c). So in this particular case, epigenetic state inherited via the male germline appears to have a greater effect, *in trans*, on the establishment of the *A*^*vy*^ phenotype. In line with our observations, it has recently been reported that ERV elements showed higher levels of derepression in male than in female *Setdb1* knockout primordial germ cells (PGCs)^31^. Furthermore, data collected from a number of *MommeD* mutant lines, supports the idea that dosage of epigenetic modifiers is more critical in male gametes than female gametes (Table 1). It was not possible to investigate for maternal effects of *Trim28*^*MD9*^ because of reduced fertility of the heterozygous females^35^.

### No global alteration in *Setdb1* ^*MD13*/+^ sperm DNA methylation

**Figure 3:**
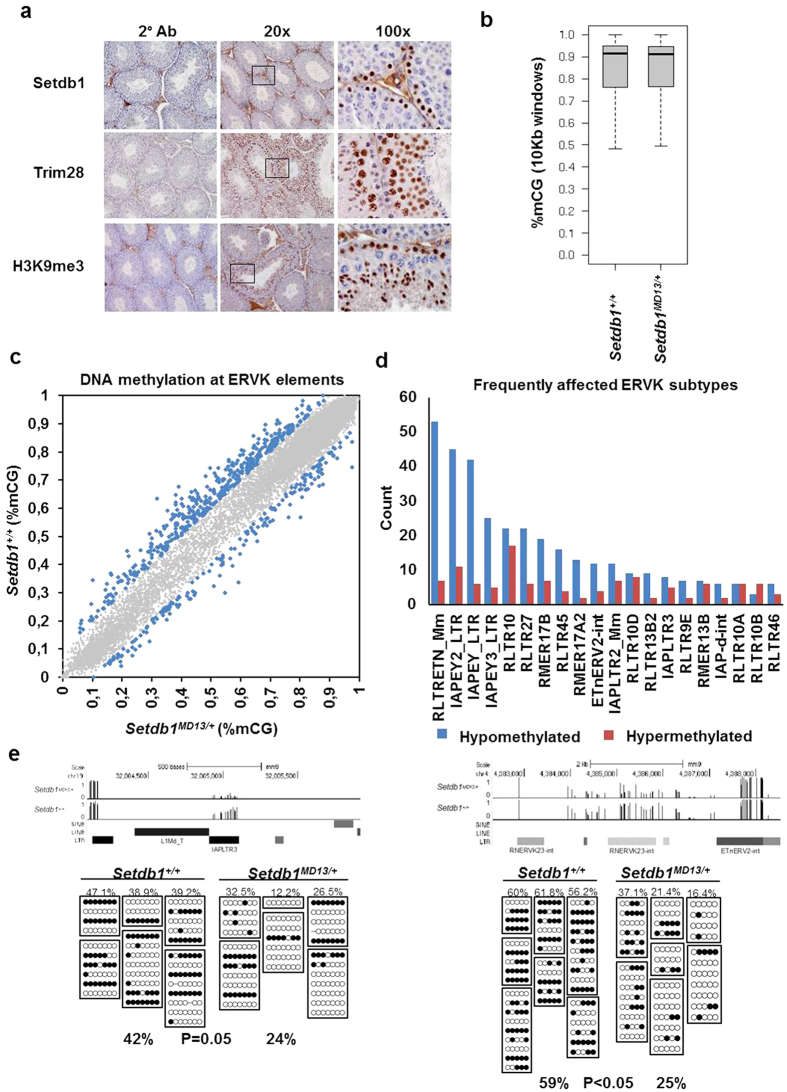
Hypomethylated regions in *Setdb1*^*MD13*/+^ correspond to ERVK elements. (**a**) Detection of Setdb1, Trim28 and H3K9me3 by immunohistochemistry in testes from 3 months old males. For each, a secondary antibody control (2°Ab) is shown to indicate the specificity of the immunohistochemical staining (first panel of each row), followed by specific staining at 20x and 100x magnification. Setdb1 was found to be present in spermatogonia, Trim28 in spermatocytes and spermatids and H3K9me3 in spermatogonia and spermatids. (**b**) Box plot of the average DNA methylation in 10Kb windows across the genome (n = 246, 281), excluding outliers (1.5× interquartile range). (**c**) Plot showing the weighted average DNA methylation for sequence elements annotated as ERVK in sperm from *Setdb1*^*MD13*/+^ and *Setdb1*^+/+^ mice (n = 16,331). Elements for which the DNA methylation difference was greater than 10 percentage points are colored blue (n = 607). (**d**) Counts of ERVK subfamilies that were either hypermethylated or hypomethylated in *Setdb1*^*MD13*/+^ relative to *Setdb1*^+/+^ and for which DNA methylation differed by more than 10 percentage points. The 20 most frequently represented subfamilies are shown. (**e**) UCSC genome browser screen capture showing a reduction of mCG at an IAPLTR3 (top left panel) and a RNERVK23-int (top right panel) in *Setdb1*^*MD13*^ heterozygotes. Sanger sequencing of bisulphite converted sperm genomic DNA from independent males confirmed reduced mCG levels of the IAPLTR3 (bottom left panel; T-test P = 0.05) and the RNERVK23-int (bottom right panel; T-test P < 0.05) in *Setdb1*^*MD13*^heterozygotes. Each boxed block of lines comprises clones derived from one bisulphite conversion and each column represents one individual with the percentage of methylated CpGs indicated above each individual. Open circles indicate an unmethylated CpG, and closed circles a methylated CpG.

The molecular mechanisms underlying epigenetic inheritance across generations in mammals remains unclear. We reasoned that an altered epigenetic state must be present in the sperm of *Setdb1*^*MD13*/+^ mice that can influence the coat colour ratios of wild type offspring and lead to the paternal effect. First, we assessed the location of Setdb1 and Trim28 protein in testis from three months old adult mice by immunohistochemistry. Setdb1 was present in spermatogonia (Fig. 3a top panel). In contrast, we detected Trim28 in spermatocytes and in round spermatids (Fig. 3a middle panel), consistent with a previous report^36^. We also assessed the location of the repressive mark H3K9me3 and found that it was present in spermatogonia but absent in mature sperm, suggesting that it is unlikely to be the transmitted mark (Fig. 3a bottom panel).

Next, we analysed DNA methylation patterns of sperm from *Setdb1*^*MD13*^ wild types and heterozygotes. We performed whole genome bisulphite sequencing to ~30-fold coverage of DNA from adult sperm from *Setdb1*^+/+^ and *Setdb1*^*MD13*/+^ mice. The resulting datasets were normalized for library size (read count), mapped to the mouse genome (mm9) and DNA methylation levels for CpG dinucleotides were calculated (see Methods). Hypomethylation of imprinting control regions confirmed the purity of the sperm samples (Supplementary Fig. 2). First, we sought to determine if there were global DNA methylation differences. We calculated the average DNA methylation in 10kb windows across the genome and found that DNA methylation levels were similar with a median of 90% (Fig. 3b).

### Hypomethylated loci are enriched for specific classes of transposable elements in *Setdb1* ^*MD13*/+^ sperm

Because the *A*^*vy*^ phenotype is defined by the epigenetic state of an IAP element, and Setdb1 has been implicated in the silencing of these, we were interested to see if there were local differences in the DNA methylation of these elements in sperm from wild types compared to *Setdb1*^*MD13*^ heterozygotes. We therefore determined the DNA methylation levels for individual repeat elements annotated as ERVK in the UCSC Genome Browser by calculating the weighted average of CpGs within each element, including only those CpGs with sufficient read-coverage and with similar coverage between the individuals (see Methods). A scatterplot of the 16,331 elements for which methylation values could be obtained revealed a skew towards decreased methylation in the sperm from mutants at some elements (Fig. 3c). Focussing on the subset of 607 elements with a difference >10 percentage points 425 elements were hypomethylated and 182 elements hypermethylated in sperm from *Setdb1*^*MD13*^ heterozygotes. The shift was significant with p-value < 2.2e-16 (Mann–Whitney U test). The differential methylation was observed most frequently for ERVK subtypes annotated as RLTRETN and IAPEY (Fig. 3d). Of the 425 elements with reduced DNA methylation levels, 53 were within 10Kb of a RefSeq transcription start site (data not shown). We used Sanger bisulphite sequencing on sperm DNA isolated from independent *Setdb1*^+/+^ and *Setdb1*^*MD13*/+^ animals to validate some of the repeat DMRs. We detected lower amounts of DNA methylation at a RNERVK23-int element in *Setdb1*^*MD13*/+^ animals (26%, T-test P < 0.05) compared to wild types (59%) (Fig. 3e). A similar trend (T-test P = 0.05) was observed for an IAPLTR3 element (42% methylated in wild types and 24% methylated in *Setdb1*^*MD13*/+^ mice) (Fig. 3e). Reduced DNA methylation was found at two out of three additional loci tested (Supplementary Fig. 3).

To investigate if the local effects at ERVK elements could also be observed at other repeats a similar analysis was performed on the two most abundant repeat classes in the mouse genome, the LINE1 and B1 elements, as well as on a set of randomly selected 1kb regions across the mouse genome (Supplementary Fig. 4). No difference in the distribution of sites with a methylation difference greater than 10 percentage points were detectable in these data sets (Mann–Whitney U test p > 0.2).

The paternal effect observed on wild type offspring of a *Setdb1*^*MD13*/+^ sire most likely occurs as a result of epigenetic events in the sire. However, the affected locus, *A*^*vy*^, is inherited via the maternal set of chromosomes. This implies a *trans* effect sometime after fertilization. Intriguingly, it has been shown that zygotic transcription of *Setdb1* does not begin until the blastocyst stage, and that Setdb1 exists as a maternal stock during preimplantation development^37^. Our data suggests that an altered epigenetic state present in the paternal genome cannot be rescued by maternal stocks of Setdb1 protein during preimplantation development. This supports a model in which hypomethylation of repetitive elements (including IAPs) or other, yet unknown Setdb1 targets in wild type sperm generated from *Setdb1*^*MD13*^ heterozygotes may adversely affect epigenetic reprogramming at *A*^*vy*^ after fertilization.

## Discussion

Our study aimed to identify factors required for establishment of epigenetic state and epigenetic inheritance at *A*^*vy*^. Overall, our results suggest that the dosage of some factors involved in epigenetic reprogramming is critical to normal epigenetic reprogramming across generations. Haploinsufficiency appears more likely to affect offspring phenotype if passed through the male gamete.

In total, we have now identified four paternal effect genes in the mouse, *Setdb1*, *Dnmt1*, *Snf2h* and *Dnmt3l*. If *A*^*vy*^ is used as the reporter locus for paternal effects, which has now been carried out for *Setdb1*, *Dnmt1* and *Snf2h*, the mechanism involves an *in trans* step (i.e. affects a maternally transmitted locus). We found DNA hypomethylation of repetitive elements in adult sperm of *Setdb1*^*MD13*^ heterozygotes. However, whether this hypomethylated state indeed is causative for the *in trans* effect or whether multiple molecular pathways that can act at specific developmental stages are involved remains to be determined. For example, in *C. elegans*, small RNA molecules and histone modifications have been shown to be required for transgenerational epigenetic inheritance^10^,^11^,^12^,^38^. It can be envisioned that similar mechanistic processes might also be involved in mammals.

Although we have revealed a critical role for Setdb1 dosage in the male germline, the actual time-point at which the paternal effect is established is unknown. Between E8.5 and E13.5 during germ cell development, most DNA methylation is lost. Subsequently, between E13.5 and birth, the genome undergoes global *de novo* DNA methylation^39^,^40^,^41^. Liu *et al.* recently reported a crucial role for Setdb1 in the silencing of ERV families, including IAP elements, in the prenatal germline prior to the onset of *de novo* DNA methylation^31^. We favour a model whereby DNA hypomethylation in adult sperm results from events during primordial germ cell (PGC) reprogramming during fetal testis development. Indeed, there is evidence that H3K4 methylation antagonizes *de novo* DNA methylation^42^ by blocking DNMT3A/3L binding to histone H3^43^,^44^ and transcriptional up-regulation of ERVK repeat family members has been reported in *Setdb1* depleted PGCs^31^. Importantly, modifications to the epigenome before the segregation of homologous chromosomes into haploid gametes could affect chromosomes entering both wild type and mutant gametes, and those differences may be retained. Further work will be required to explore these possibilities.

Trim28 has been shown to be involved in transposon repression in the early embryo^30^,^45^ and consistent with this observation, we find a shift towards a higher percentage of yellow mice in the offspring that inherit the Trim28 mutation. However, we did not observe a paternal effect. As mentioned previously, it is currently unclear when the paternal effect is established and it is possible that this occurs at a time point when Trim28 is dispensable or reduced dosage can be compensated for by other factors.

Surprisingly, we found that maternal haploinsufficiency for Setdb1 has no effect on offspring coat colour phenotype. It has previously been shown that DNA methylation levels at the blastocyst stage differ, depending on whether the *A*^*vy*^ allele was inherited from the father (21% methylation) or the dam (0% methylation)^34^. It could be that in our crosses between Setdb1 haploinsufficient females and *A*^*vy*^ males pre-existing DNA methylation at the *A*^*vy*^ locus, inherited from the male gametes, promotes silencing of the locus in a Setdb1 independent manner.

In conclusion, while our studies have used the *A*^*vy*^ locus as a sensitive reporter, there are likely to be other regions in the genome that are sensitive to dosage in a similar way and behave like metastable epialleles. There is increasing evidence for complex diseases associated with mutations in genes coding for chromatin proteins and our findings suggest that epigenetic inheritance across generations may be more likely to occur in these families.

## Methods

### Mouse strains and genotyping

All experiments were performed in accordance with the Australian Code of Practice for the care and use of animals for scientific purposes and the Dutch Animals Act. Procedures were approved by the Animal Ethics Committee of the QIMR Berghofer Medical Research Institute, La Trobe University Melbourne or Leiden University Medical Center and by the Commission Biotechnology in Animals of the Dutch Ministry of Agriculture. The ENU screen was carried out in the FVB/NJ inbred transgenic line, Line3, as described previously^33^. The resultant *MommeD* mutant lines were maintained in this background. Mice carrying the *A*^*vy*^ allele were maintained on a C57BL/6J background. Primer sequences for genotyping for the *A*^*vy*^ allele and the *MommeD* mutations are provided in Supplementary Table S1. *Trim28*^*MD9*^ mice were classed as heterozygous or wild type by their GFP expression profile using flow cytometry as described previously^35^.

### Crosses between *MommeD* mutants and mice carrying the *A* ^*vy*^ allele

Coat colour phenotype of F1 offspring was classified at weaning by a trained observer blind to genotype (with respect to the *MommeD* mutation). The percentage of yellow, mottled and agouti (brown) coat colour on each mouse was determined and the mouse was placed into one of five categories: yellow (100% yellow), yellow^mottled^ (>95% yellow), mottled (<95% yellow and >25 yellow), pseudoagouti^mottled^ (between 75–95% agouti) or pseudoagouti (>95% agouti, ψ). These were then pooled into three groups (Y – yellow and yellow^mottled^, M- mottled or ψ- pseudoagouti^mottled^ and pseudoagouti). At least five different breeding pairs were set up for each cross. The proportion of genotypes were compared to expected Mendelian ratios using a χ^2^ test.

### Sperm isolation and DNA extraction

Mature spermatozoa were squeezed from perforated cauda epididymis and vas deferens into 2 ml Dulbecco’s Modified Eagle Medium (DMEM) in a petri dish and mixed by pipetting. The suspension was transferred to a 12 ml falcon tube and left upright for 20 min. Thereafter the top 1.5 ml was transferred to two 1.5 ml Eppendorf tubes and stored on ice. Sperm were pelleted by centrifugation for 1 min at 8000 rpm. Sperm were washed and re-pelleted once with PBS. Any contaminating somatic cells were removed with resuspension in two solutions, firstly a wash in distilled water for osmotic lysis, secondly, sperm were incubated for 20 min on ice in somatic cell lysis buffer (0.1% SDS, 0.5% Triton-X). Lysates were stored at −80 until further use. Genomic DNA was isolated using the DNeasy Blood & Tissue Kit (Qiagen, Doncaster, VIC, Australia) according to protocol with the exception of the addition of 16ul 1M DTT 5min before addition of buffer AL to aid lysis of the sperm, which are more compact than somatic tissue.

### Whole Genome Bisulphite sequencing

Whole Genome Bisulphite Sequencing was performed by the Centro Nacional de Analisis Genomice (CNAG, Barcelona, Spain). Genomic DNA samples were spiked with lambda DNA and sheared by sonication. Libraries were prepared using the TruSeq Sample Prep Kit (Illumina, San Diego, CA) and underwent two rounds of sodium bisulphite conversion using the EpiTect Bisulphite Kit (Qiagen, Doncaster, VIC, Australia). 100 bp paired end sequencing was performed on the Illumina HiSeq 2000. The sequencing reads were initially processed by trimming low quality 3′ ends using the Biopieces tool trim_seq (www.biopieces.org) with the options -trim = right -l 3 -m 15. Resulting reads were mapped to the mouse genome (mm9) using the program Bismark (version 0.13.0) with the options -bowtie2 -N 1 -D 100 -R 10 -maxins 300. The number of mapped reads for the two libraries were equalized by randomly subsampling the larger of the two using samtools with the option -s 0.85. The mapped reads were then processed by the Bismark tool deduplicate_bismark to remove PCR duplicates, filtered for poorly mapped reads by running samtools view with the option -q 38 and DNA methylation values obtained for each strand using the Bismark tool bismark_methylation_extractor with the options –ignore_r2 2 –counts –paired-end –no_overlap –merge_non_CpG. DNA methylation calls from the two strands were finally merged using custom scripts to obtain a single methylation value for each CpG dinucleotide.

DNA methylation differences at repetitive elements (repeat annotations were obtained from the RepeatMasker track in the UCSC Genome Browser, mm9 assembly) were identified by first eliminating those CpGs with <8 reads coverage in either sample. Sites with a bias in read coverage between samples were also removed according to the rule that the sample with the lowest coverage must be within 60% of the coverage in the other sample. For those repeats with at least 8 CpGs satisfying the previous criteria, the weighted average was calculated by dividing each CpG score by the sum of read-counts for the region, multiplying each CpG by its own read count and finally adding together each adjusted score in the region to get the final weighted score.

### Sanger bisulphite sequencing

1ug of genomic DNA was converted using the Zymo lightning Kit (Zymo Research Corp., Orange, CA, USA) according to the manufacturer’s instructions. The bisulphite conversion rate was at least 97% and sequences were analysed using the BiQ Analyser software^46^. A list of oligonucleotides is provided in Supplementary Table 1. Cycling conditions were as follows: 94 °C for 2min for 1 cycle; 94 °C for 30 seconds, 60 °C for 30 seconds, 72 °C for 45 seconds for 35 cycles and 72 °C for 6 minutes for 1 cycle.

### Protein analysis

Whole cell extract was prepared from adult testes (three months old males) by homogenising in ten volumes of urea lysis buffer, as described^32^. Samples were measured by BCA (Thermo Scientific, Waltham, MA, USA) and lysates separated on a polyacrylamide gel (BioRad, Hercules, CA, USA). Antibodies used were as follows: anti-Setdb1 antibody (11231-1-AP Protein Tech, Chicago, IL, USA) and anti-gamma-tubulin (T5192, Sigma-Aldrich, St. Louis, MO, USA).

### Immunohistochemistry

Adult testes from 3 months old males were collected and fixed in Bouin’s fixative then stored overnight in ethanol before processing in xylene and ethanol to be embedded in paraffin wax. Sections were cut at 5 μm and baked onto slides at 60 °C. Vectastain Elite ABC Kit (Universal) (Vector Laboratories, Burlingame, CA, USA) was used to detect protein after heat induced antigen retrieval. Overnight incubation in primary antibodies anti-Setdb1 antibody (11231-1-AP, Proteintech, Chicago, IL, USA) at 1:200, anti-Trim28 antibody (MP-2365522, Merck-Millipore) at 1:1500 and anti-histone H3 (trimethyl K9) (ab8898, Abcam) at 1:400 at 4 °C was followed by secondary biotinylated universal antibody (BA-1400, Vector Laboratories) incubation and ABC reagent incubation before development of DAB stain (SK-4100, Peroxidase substrate Kit, Vector Laboratories).

## Additional Information

**Accession codes:** Raw whole-genome bisulphite-sequencing data generated for this study have been deposited in the GEO database under accession number GSE72265.

**How to cite this article**: Daxinger, L. *et al.* Hypomethylation of ERVs in sperm of mice haploinsufficient for the histone methyltransferase Setdb1 correlates with a paternal effect on phenotype. *Sci. Rep.* **6**, 25004; doi: 10.1038/srep25004 (2016).

## Supplementary Material

Supplementary Information

## Figures and Tables

**Table 1:** Effect of parental haploinsufficiency of epigenetic modifiers on expression of *A*^*vy*^in offspring.

	Mutant sire (Maternally derived *A*^*vy*^ allele)		Mutant dam (Paternally derived *A*^*vy*^ allele)		References
	Effect on wild-type offspring	Effect on mutant offspring	Effect on wild-type offspring	Effect on mutant offspring	
*Dnmt1*^*MD2*^	Yes	Yes	No	Yes*	Blewitt *et al.*^33^, Chong *et al.*^24^ and data not shown
*Snf2h*^*MD4*^	Yes	Yes	n.d.	n.d.	Chong *et al.*^24^
*Rlf *^*MD8*^	n.d.	n.d.	No	Yes	Daxinger *et al.*^32^
*Smchd1*^*MD1*^	No	Yes	No	No	Blewitt *et al.*^33^
*Trim28*^*MD9*^	No	Yes	n.d.	n.d.	This study
*Setdb1*^*MD13*^	Yes	Yes	No	No	This study

*Female specific effect.
