# Supplementary material for: Hypomethylation of ERVs in the sperm of mice haploinsufficient for the histone methyltransferase Setdb1 correlates with a paternal effect on phenotype
Source: Sci Rep. 2016 Apr 26;6:25004. doi: 10.1038/srep25004 (PMC4845014; doi:10.1038/srep25004)
Supplement: Supplementary Information [file srep25004-s1.pdf]

SUPPLEMENTARY INFORMATION

Hypomethylation of ERVs in the sperm of mice haploinsufficient for the histone methyltransferase Setdb1 correlates with a paternal effect on phenotype

Lucia Daxinger[1,2], Harald Oey[1,*], Luke Isbel[1], Nadia C. Whitelaw[3], Neil A. Youngson[4], Alex Spurling[1], Kelly Vonk[2], Emma Whitelaw[1,3]

[1]Department of Genetics, La Trobe Institute for Molecular Science, La Trobe University, Bundoora, VIC, 3086 Australia

[2]Department of Human Genetics, Leiden University Medical Centre, Leiden, The Netherlands

[3]Epigenetics Laboratory, QIMR Berghofer Medical Research Institute, Herston, Brisbane 4006, QLD, Australia

[4]Department of Pharmacology, School of Medical Sciences, UNSW Australia, Sydney 2052, Australia

*Present address: University of Queensland Diamantina Institute, Translational Research Institute, Princess Alexandra Hospital, Brisbane, QLD 4102, Australia

Correspondence should be addressed to L.D. (email: l.clemens-daxinger@lumc.nl) or to E.W. (email: e.whitelaw@latrobe.edu.au).

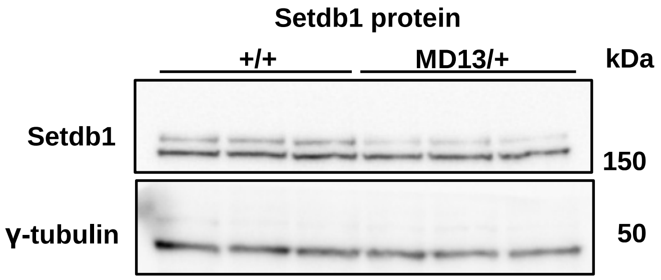

Supplementary Figure 1. Western blot showing reduced levels of Setdb1 in adult testes. Western blot analysis of testes from 3 months old males shows that *Setdb1^{MD13}* heterozygotes have a reduced dosage of Setdb1. Each track represents a different animal. Setdb1 protein was detected at approximately 170 kDa (upper band) and tubulin was used as a loading control.

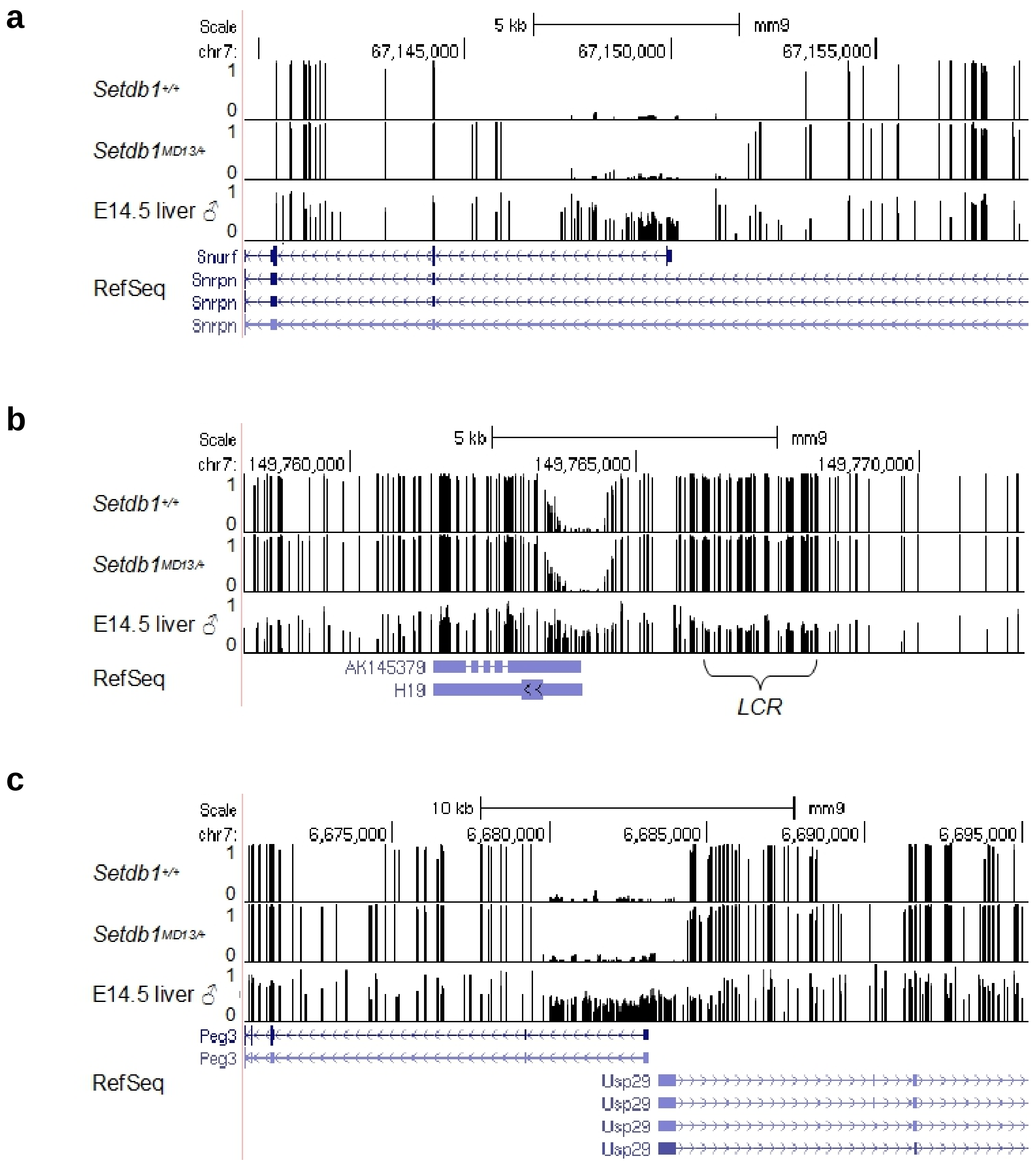

Supplementary Figure 2. Sperm DNA methylation profiles for a selection of imprinted loci. DNA methylation profiles of the locus control regions (LCR) for three imprinted loci are shown. Each vertical bar represents the DNA methylation at a CpG dinucleotide. Only those CpGs with at least 6 read coverage are shown and a value of 0 represents unmethylated and 1 represents fully methylated. The imprinted loci are (**a**) the paternally expressed Snurf/Snrpn locus (expected to be unmethylated in sperm), with LCR at the promoter of the SNURF gene, (**b**) the maternally expressed H19 locus (expected to be methylated in sperm), with LCR at the H19 promoter and (**c**) the maternally expressed Peg3 locus (expected to be unmethylated in sperm), with location of the LCR as indicated. Methylation profiles from a publicly available somatic tissue (GEO accession GSM1400928), which is expected to be 50% methylated at the LCRs, are also shown. Contamination of sperm DNA with DNA from somatic cells would be expected to cause the sperm DNA methylation values to approach somatic levels.

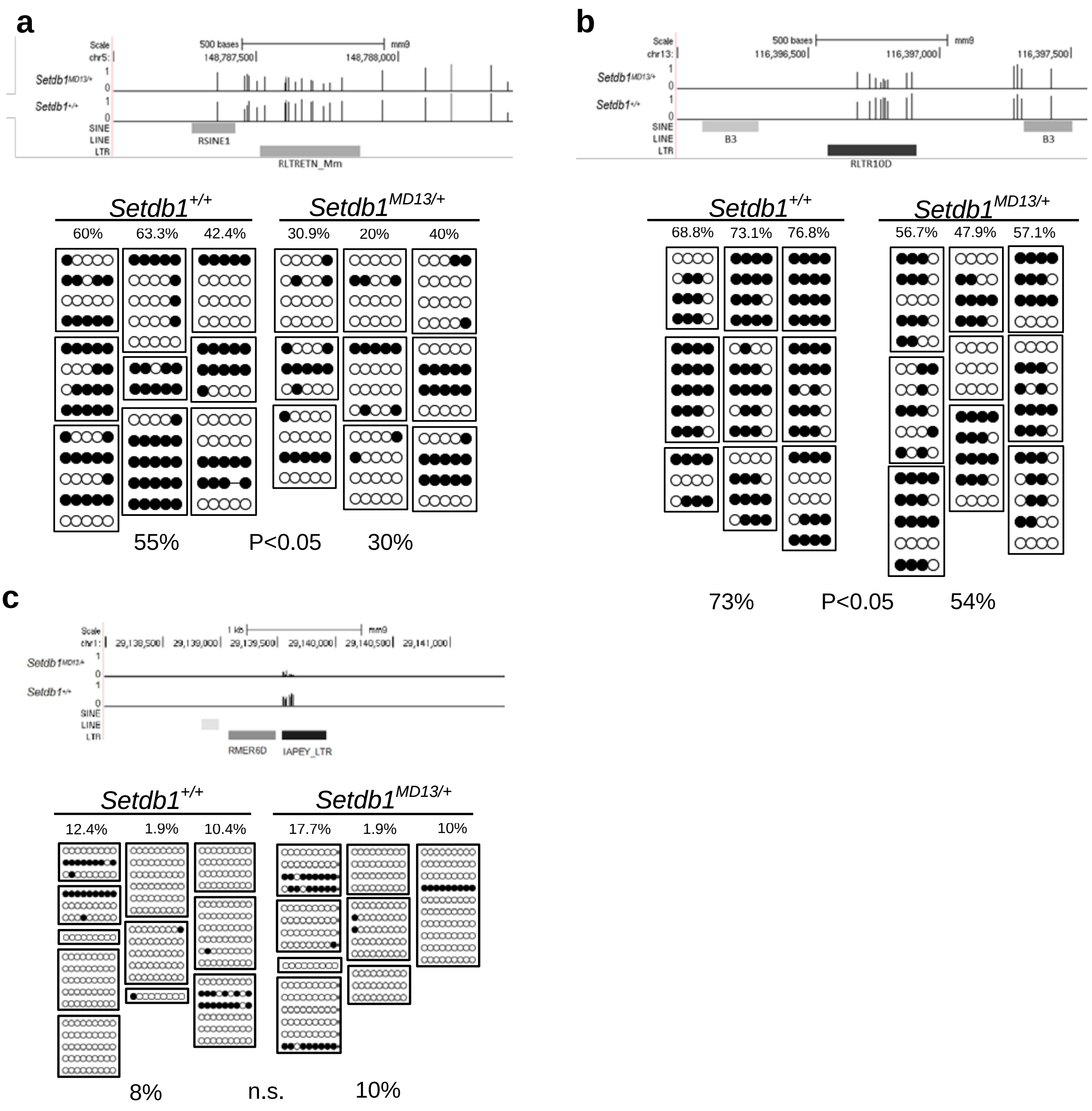

Supplementary Figure 3: Validation of repeat hypomethylation in *Setdb1*^{MD13} sperm by Sanger bisulphite sequencing. (**a**) UCSC genome browser screen capture showing a reduction of mCG at an RLTRETN element in *Setdb1*^{MD13} heterozygotes (top panel). Sanger sequencing of bisulphite converted sperm genomic DNA from independent males confirmed reduced mCG levels in *Setdb1*$^{MD13/+}$ males (T-test P<0.05) (bottom panel). (**b**) Screen capture showing hypomethylation of an RLTR10D element in *Setdb1*$^{MD13/+}$ sperm (top panel). Reduced DNA methylation levels were found in independent *Setdb1*$^{MD13/+}$ males (T-test P<0.05) (bottom panel). (**c**) UCSC snapshot demonstrating reduced mCG levels at an IAPEY_LTR element in *Setdb1*^{MD13} heterozygotes (top panel). No differences in mCG levels between wild types and *Setdb1*$^{MD13/+}$ mutants were detected in independent males.

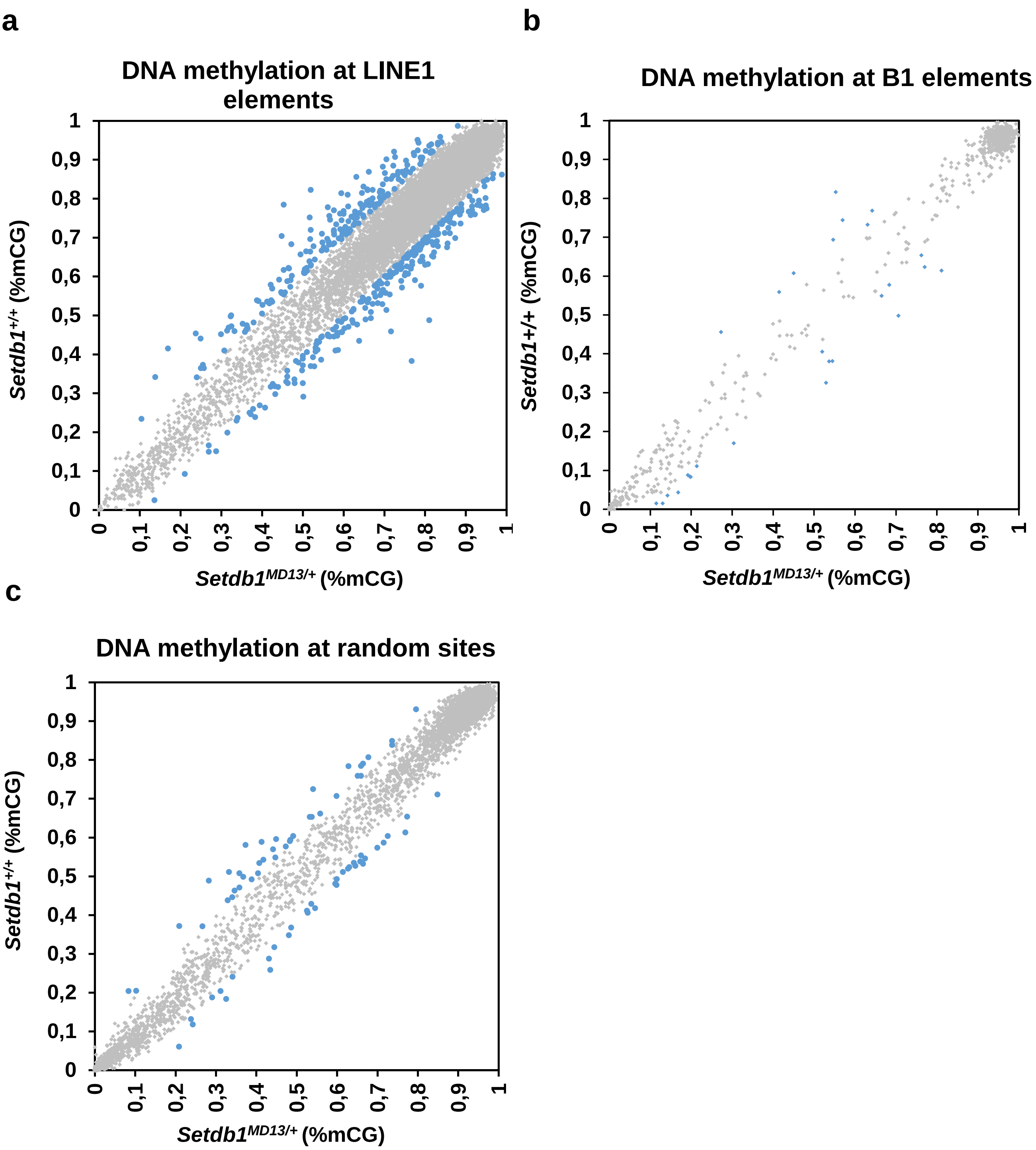

Supplementary Figure 4. Comparison of DNA methylation levels in *Setdb1*$^{+/+}$ and *Setdb*$^{MD13/+}$ sperm at selected sites in the genome. The weighted averages for the DNA methylation of CpGs at selected sites in the mouse genome are shown. Only sites satisfying the parameters outlined in the methods section are plotted and sites with a difference in DNA methylation >10 percentage points are highlighted in blue. (**a**), LINE1 elements, 16,432 sites are plotted of which 439 sites have a difference >10. (**b**) B1 elements, 823 sites are plotted of which 27 sites have a difference >10. (**c**) Random 1kb regions, 8,526 sites are plotted of which 73 sites have a difference >10.

List of primers

A^{vy}_genotyping

Agouti_5': CATGGCTACAGCATCCTGACAA
Agouti_3': TGGCCAGGAAAGAAGGAAACTG
IAP-F: GCGCATCACTCCCTGATTG

*Setdb1*_genotyping

Setdb1-F: AGTTCTATGATGCACAGTTGCAG
Setdb1-R: CTGCAACTGTGCATCATAGAACT

Bisulphite_validations

Chr19_IAPLTR3-F: ATTTATTAGTTGATTGTGAGTATTTATTTTTGTGTTTG
Chr19_IAPLTR3-R: TCAAAATATCRTTCCATTACCTAAACCCTACCTC

Chr4_RNERVK23-int-F: GTTTTTTGGGGTTGTTTATAGGTTGAGG
Chr4_RNERVK23-int-R: TATAAACAACCTAAACATTTTACCAAACAATACCCCC

Chr1_IAPEY_LTR_F: TATGGAAGTTAGAAGTTTTTAGGTTTAGTTTTAG
Chr1_IAPEY_LTR_R: ATTCACTCTCRCACCTATATACAAAACTTATTTAC

Chr13_RLTR10D_F: GTAGTTTTTAYGGTAGATGTGATTTATTTTTGGGTG
Chr13_RLTR10D_R: AATACCAAAACTCAATAAAAAAAAATAAAAACAAAAC

Chr5_RLTRN_Mm_F: TTATTATTTTYGATATTTAGTTATGAAGGAGGGGAAG
Chr5_RLTRN_Mm_F: AAAAAATAATTACCCTTACCCTCCCTAAATATAACC

Supplementary Table 1. List of Primers
